# Glances at the Hospitals

**Published:** 1904-12-03

**Authors:** 


### GLANCES AT THE HOSPITALS.

### SCARBOROUGH HOSPITAL.

The total income from all sources was £2,508, and the total expenditure was £2,626, showing a balance of £117 on the wrong side. The board appeal to the numerous friends of the hospital for assistance to enable them to maintain the high state of efficiency of the hospital, and it is to be hoped that this appeal will not be made in vain. We are pleased to note that the fire appliances have been examined and improved lately. Fire-alarm bells have been placed in various places, and now connect distant parts of the building with the entrance hall. The in-patients numbered 426, and the average stay of each patient in the hospital was 19½ days. The medical and surgical tables show the numbers of patients treated for the various diseases and also give the number of deaths in each disease; but when we come to the operation table we get no results, merely the numbers of patients operated on. An anæsthetic table is given, and from it we learn that in the 210 operations, ether was the agent in 112, chloroform in 70, nitrous oxide in 7, and A.C.E. mixture in 21.

